# GD2 expression in breast cancer

**DOI:** 10.18632/oncotarget.16363

**Published:** 2017-03-18

**Authors:** Giulia Orsi, Monica Barbolini, Guido Ficarra, Giovanni Tazzioli, Paola Manni, Tiziana Petrachi, Ilenia Mastrolia, Enrico Orvieto, Carlotta Spano, Malvina Prapa, Shaniko Kaleci, Roberto D’Amico, Valentina Guarneri, Maria Vittoria Dieci, Stefano Cascinu, Pierfranco Conte, Federico Piacentini, Massimo Dominici

**Affiliations:** ^1^ Department of Medical and Surgical Sciences for Children and Adults, University-Hospital of Modena and Reggio Emilia, 71-41124 Modena, Italy; ^2^ Division of Pathology, University-Hospital of Modena and Reggio Emilia, 71-41124 Modena, Italy; ^3^ Breast Unit, University-Hospital of Modena and Reggio Emilia, 71-41124 Modena, Italy; ^4^ Department of Pathology, Padua University Hospital, 2-35128 Padua, Italy; ^5^ Department of Diagnostic and Clinical Medicine and Public Health, Statistics Unit, University-Hospital of Modena and Reggio Emilia, 71-41124 Modena, Italy; ^6^ Department of Surgery, Oncology and Gastroenterology, Division of Medical Oncology 2, Istituto Oncologico Veneto IRCCS, Via Gattamelata, 64-35128 Padua, Italy

**Keywords:** GD2, disialoganglioside, BC, TNBC, metaplastic

## Abstract

Breast cancer (BC) is a heterogeneous disease, including different subtypes having diverse incidence, drug-sensitivity and survival rates. In particular, claudin-low and basal-like BC have mesenchymal features with a dismal prognosis. Disialoganglioside GD2 is a typical neuroectodermal antigen expressed in a variety of cancers. Despite its potential relevance in cancer diagnostics and therapeutics, the presence and role of GD2 require further investigation, especially in BC. Therefore, we evaluated GD2 expression in a cohort of BC patients and its correlation with clinical-pathological features.

Sixty-three patients with BC who underwent surgery without prior chemo- and/or radiotherapy between 2001 and 2014 were considered. Cancer specimens were analyzed by immunohistochemistry and GD2-staining was expressed according to the percentage of positive cells and by a semi-quantitative scoring system.

Patient characteristics were heterogeneous by age at diagnosis, histotype, grading, tumor size, Ki-67 and receptor-status. GD2 staining revealed positive cancer cells in 59% of patients. Among them, 26 cases (41%) were labeled with score 1+ and 11 (18%) with score 2+. Notably, the majority of metaplastic carcinoma specimens stained positive for GD2. The univariate regression logistic analysis revealed a significant association of GD2 with triple-receptor negative phenotype and older age (> 78) at diagnosis.

We demonstrate for the first time that GD2 is highly prevalent in a cohort of BC patients clustering on very aggressive BC subtypes, such as triple-negative and metaplastic variants.

## INTRODUCTION

Breast cancer (BC) survival rates have increased in the last decades, thanks to intense screening programs and to increasingly targeted and effective therapeutics [1]. BC is a heterogeneous disease with different patterns of incidence, survival and drug-sensitivity. In the last few years gene expression profiling identified five molecular intrinsic subtypes. In the early 2000 luminal A, luminal B, Her2-enriched and basal-like BC were described [2–4]. Later, Prat et al. defined a claudin-low subtype, characterized by low expression of genes involved in cell-cell adhesion, high mesenchymal features and with stem cell features [5]. While the luminal A and B can be mostly identified by estrogen and progesterone receptors and the Her2-enriched subtypes by a HER2 positivity, the highly aggressive basal-like and claudin-low tumors are included in the triple negative BC (TNBC) phenotype and still lack of more defined markers [4, 5]. Moreover Lehmann et al. identified six TNBC subtypes displaying unique gene expression (basal-like 1 and 2, immunomodulatory, mesenchymal, mesenchymal stem-like and luminal androgen receptor) capable to inform therapy selection [6].

In this perspective we focused on disialoganglioside GD2, a surface antigen, primarily expressed in the nervous system and skin melanocytes as well as in the stromal component of other normal tissues [7]. More recently, we have identified GD2 as a surface marker of mesenchymal stromal cells (MSC), adult progenitors that can be isolated from different tissues and *ex vivo* expanded [8]. In cancer, disialoganglioside GD2 is typically expressed in neuroectodermal tumors, such as neuroblastoma, glioma and melanoma [9, 10], but it can also be found in a variety of sarcomas [11–13].

Battula et al. identified GD2 as a suitable marker for BC stem cells (BCSC) and its expression was described in BC cell lines as well as in a few histological samples [14]. Moreover, the inhibition of GD3 synthase, the critical enzyme involved in GD2 synthesis, has been shown to prevent metastasis in in-*vivo* mouse models, to inhibit epithelial-mesenchymal transition (EMT) and to compromise mesenchymal characteristics of claudin-low BC cell lines [14–16]. Despite the relevance of these data, the possible role of GD2 as a BC biomarker and therapeutic target still requires in-depth investigations. For this reason, we sought to report for the first time histological evidences of the GD2 expression pattern in a heterogeneous cohort of BC samples, highlighting the peculiar tendency of this antigen to correlate with a mesenchymal-like phenotype and significantly with the triple negative phenotype.

## RESULTS

### Patients

Clinical-pathological characteristics of the sixty-three patients included in the study are shown in Table 1. Average age at diagnosis was 67 ± 15 years old. The most represented histological types were invasive ductal carcinoma (IDC) not otherwise specified (NOS) (37/63; 59%), metaplastic carcinoma (14/63; 22%) and invasive lobular carcinoma (ILC) (7/63; 11%). The other cases included mixed carcinoma (IDC + invasive lobular carcinoma) (2/63; 3%), apocrine ductal carcinoma (2/63; 3%) and one sample of carcinosarcoma (2%). Concerning the histological grade, in most cases (49/63; 78%) tumors were G3; the other cases (14/63; 22%) were G2.

**Table 1:** Patient and tumor characteristics

Clinical-pathological parameters	N°/63 (%)
**Median age at diagnosis ± SD**	67 ± 15 yrs
**Histological Type**	
IDC	37 (59)
ILC	7 (11)
Mixed (IDC+ILC)	2 (3)
Metaplastic	14 (22)
Apocrine	2 (3)
Carcinosarcoma	1 (2)
**Histological Grade**	
**G2**	14 (22)
G3	49 (78)
**Tumor Size – pT**	
pT1	27 (43)
pT2	22 (35)
pT3	11 (17)
pT4	3 (5)
**Hormone Receptor and HER2 status**	
ER/PR +, HER2 –	18 (29)
ER/PR -, HER2 +	2 (3)
ER/PR +, HER2 +	3 (5)
TNBC	40 (63)
**Ki-67**	
< 20%	21 (33)
> 20%	42 (67)

IDC = invasive ductal carcinoma; ILC = invasive lobular carcinoma; ER = estrogen receptor; PR = progesteron receptor; TNBC = triple-negative breast cancer

Tumor size ranged from 0,7 cm to 10 cm, with a mean of 3,2 ± 2,4 cm with 42 (67 %) patients having a Ki-67 expression greater than 20%. Twenty-seven samples (43%) were pT1, 22 (35%) were pT2, 11 (17 %) were pT3 and three samples were pT4 (5%). Forty patients (63%) were both hormone receptors and HER2 negative (TNBC). Eighteen cases (29%) were hormone receptors positive, but did not over-expressed HER2. Five samples over-expressed HER2: 3 (5%) were also hormone receptor positive, 2 (3%) were ER/PR negative.

### GD2 marks BC cells clustering on TNBC

The evaluation of GD2 IHC staining in the sixty-three BC specimens revealed an antigen expression in 37 cases (59%).

**Figure 1:**
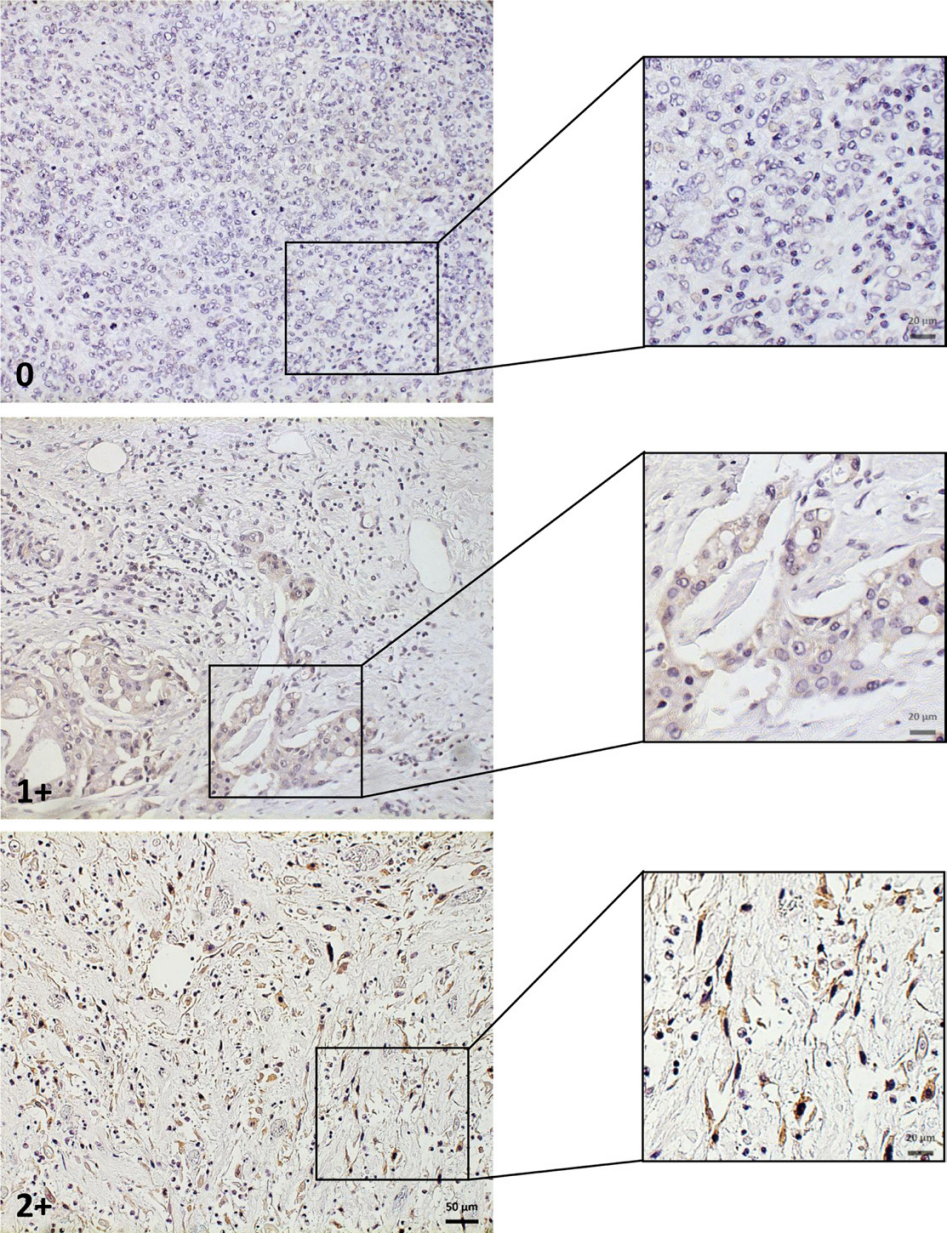
GD2 scoring system Representative areas of the different GD2 staining intensities are shown at greater magnification in insets. Scale bar: 50 μm.

**Figure 2:**
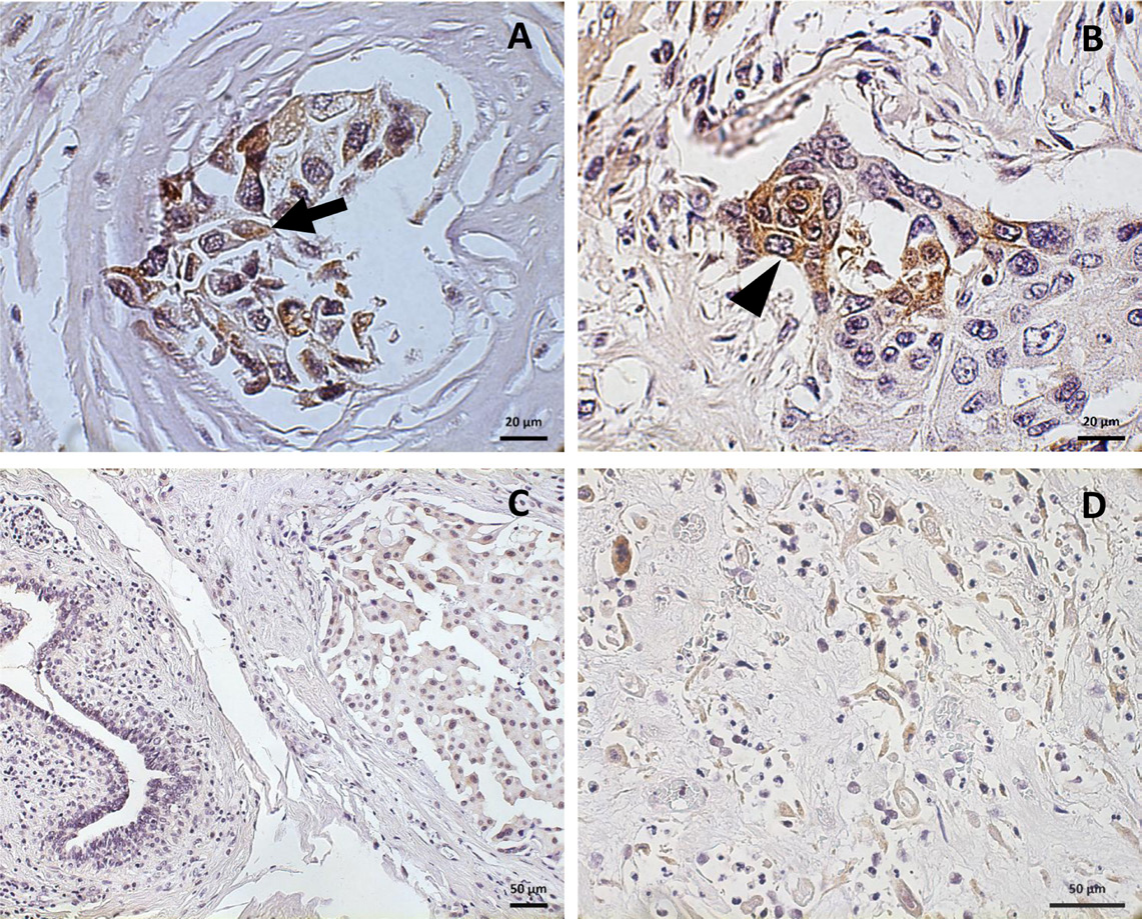
Pattern of GD2 localization in breast cancer Cytoplasmatic (**A**; arrow) and membrane (**B**; arrow head) positivity for GD2 in breast cancer specimens. Few GD2 positive cells are visible within negative stroma (**C**). Strong GD2 staining in spindle cells in a metaplastic sarcomatoid breast cancer section (**D**) Scale bar: 20 μm (A, B), 50 μm (C, D).

According to our GD2 semi-quantitative scoring system (Figure 1), 26 cases (41%) were negative (score 0); among the GD2 positive specimens (37/63; 59%), 26 (41%) were identified with score 1+ and 11 (18%) with score 2+. The percentage of positive cells in the GD2 expressing specimens was extremely variable, ranging from 3% to 100%, with a mean of 52% ± 30%. Having observed this positivity, we focused on the staining pattern. GD2 was mainly expressed in the cytoplasm (Figure 2A). Additionally, BC cells showed a GD2 staining reinforcement on the cell membrane (Figure 2B). Of note stroma and normal glandular ducts were apparently not stained, confirming the specificity of staining on BC cells (Figure 2C).

After observing the relevance of the staining, we then correlated the GD2 status with clinical-pathological parameters as shown in Table 2. No statistical correlation was found between GD2 and the considered variables according to Student's *t-test*, χ^2^ test and Fisher's exact test. However, the samples from metaplastic sarcomatoid carcinoma retained the highest staining, with GD2 expression mostly related to spindle cells versus epithelioid elements (Figure 2D).

**Table 2:** Correlation between GD2 expression and clinical-pathological parameters

Clinical-pathological parameters	GD2 negative N° (%)	GD2 positive N° (%)	Total	*p* value
**Patients**	26 (41)	37 (59)	63	
**Median age at diagnosis ± SD**	63 ± 14	70 ± 15		0.058
**Histological Type**				0.137
IDC	19 (51)	18 (49)	37	
ILC	4 (57)	3 (43)	7	
Mixed (IDC + ILC)	0 (0)	2 (100)	2	
Metaplastic	3 (21)	11 (79)	14	
Apocrine	0 (0)	2 (100)	2	
Carcinosarcoma	0 (0)	1 (100)	1	
**Histological Grade**				0.223
G2	8 (57)	6 (43)	14	
G3	18 (37)	31 (63)	49	
**Tumor Size – pT**				0.433
pT1	13 (48)	14 (52)	27	
pT2	9 (41)	13 (59)	22	
pT3	4 (36)	7 (64)	11	
pT4	0 (0)	3 (100)	3	
**Hormone Receptor and HER2 status**				0.158
ER/PR +, HER2 -	11 (61)	7 (39)	18	
ER/PR –, HER2 +	1 (50)	1 (50)	2	
ER/PR +, HER2 +	1 (33)	2 (67)	3	
TNBC	13 (33)	27 (67)	40	
**Ki-67**				0.205
< 20%	11 (52)	10 (48)	21	
> 20%	15 (36)	27 (64)	42	

IDC = invasive ductal carcinoma; ILC = invasive lobular carcinoma; ER = estrogen receptor; PR = progesteron receptor; TNBC = triple-negative breast cancer.

To further investigate a possible association of GD2 with any of the clinical-pathological parameters we performed both multivariate and univariate logistic regression analyses. While multivariate analysis showed no significant correlations (including Ki-67 expression), the univariate analysis (Table 3) indicated that in GD2 positive specimens the possibility of being TNBC was significantly higher than being ER/PR +/HER2– (OR = 3.26; *p* = 0.04; 95% CI [1.03–10.37]), suggesting how GD2 may represent a novel marker capable to identify a still elusive BC subtype. We also compared the GD2 expression in relationship with age, revealing a significant correlation within the older patients range (> 78) with significant higher GD2 expression (OR = 3.66; *p* = 0.04; 95% CI [1.05–12.79]).

**Table 3:** Univariate logistic regression analysis

Clinical-pathological parameters	OR	*P*	[95% CI]
**Age at diagnosis (yrs)**			
0–69	reference		
70–78	3.27	0.09	[0.83–12.81]
> 78	3.66	0.04	[1.05–12.79]
**Histological Type**			
IDC	reference		
Metaplastic	3.87	0.06	[0.92–16.17]
ILC	0.79	0.78	[0.15–4.04]
Mixed (ILC+IDC)	1	na	na
Apocrine	1	na	na
Carcinosarcoma	1	na	na
**Histological Grade**			
G2	reference		
G3	2.23	0.18	[0.69–7.68]
**Tumor Size – pT**			
pT1	reference		
pT2	1.34	0.61	[0.43–4.18]
pT3	1.62	0.51	[0.38–6.87]
pT4	1	na	na
**Hormone Receptor and HER2 status**			
ER/PR +, HER2 –	reference		
TNBC	3.26	0.04	[1.03–10.37]
ER/PR -, HER2+	1.57	0.76	[0.08–29.41]
ER/PR+, HER2+	3.14	0.38	[0.24–41.51]
**Ki-67**			
< 20%	Reference		
> 20%	1.98	0.21	[0.68–5.74]

IDC = invasive ductal carcinoma; ILC = invasive lobular carcinoma; ER = estrogen receptor; PR = progesteron receptor; TNBC = triple-negative breast cancer.

## DISCUSSION

To the best of our knowledge, in this report we describe the expression of GD2 in the largest cohort of BC so far reported. By the introduction of a new semi-quantitative IHC scoring system, we provide evidence that the majority of the analyzed samples resulted GD2 positive.

Disialoganglioside GD2 is synthesized in the endoplasmatic reticulum and Golgi's apparatus by the enzyme GD3 synthase. The antigen is then transferred on the outside layer of the plasma membrane. On the cell surface, GD2 is involved in cell-to-cell adhesion and signal transduction, playing a crucial role both in physiological (e.g. embryogenesis) and in pathological processes, such as cancer, by driving proliferation, neoangiogenesis, immune-escape and invasion. [17–19]. GD2 represents a typical marker of neuroectodermic tumors. Neuroblastoma shows a constant expression of this ganglioside, which has been exploited as a therapeutic target for monoclonal antibody and cellular therapy [7, 20–22]. GD2 was also found in tissue sections and cell lines of other neuroectodermic tumors, such as skin and uveal melanoma, small cell lung cancer and glial tumors [9, 10, 23–25]. In addition, GD2 labels soft tissue and bone sarcomas [11–13, 26, 27]. In normal conditions, although at lower levels, it is expressed in nervous tissues and in adult progenitors [8, 20], confirming its role as both neuroectodermal and mesenchymal marker.

Originally GD2 expression was investigated in BC by Battula et al., who identified this ganglioside in BC cell lines, showing positive levels between 0,1–18% with the highest expression curiously related to a basal-like phenotype [14]. The authors additionally focused on BCSC reporting GD2 as their putative biomarker. BCSC are a rare subset of cells within the tumor exhibiting a high tumorigenic potential [28–30]. BCSC have also been related to the EMT and to MSC, since the induction of EMT in mammary cells results in the acquisition of features that are typical of both BCSC and MSC [28, 29, 31, 32]. These findings were also confirmed by Liang et al. focusing on the expression profile of glycosphingolipids in human BCSC [15]. The role of GD2 as a marker of EMT and BCSC in BC was also indirectly demonstrated through the inhibition of GD3 synthase, which compromised the initiation and maintenance of EMT, as well as the mesenchymal characteristics of claudin-low BC cell lines SUM159 and MDA-MB-231 [16].

The immunohistochemical analysis of GD2 expression in our 63 specimens showed a positive staining in 59% of the cases. Battula et al. considered 12 patients with a variety of BC histotypes and, considering as an arbitrary cut-off of 5%, their positivity level seems similar (58.3%) to the one here reported in a greater BC cohort. Their positive cells ranged from 0.5 to 37.9% and, very interestingly, the highest positivity is described in the two metastatic specimens peaking in the only TNBC specimen included in the study [14]. No correlation analyses with clinical-pathological features were performed in that study, presumably due to the low number of included patients.

In this research we focused on GD2 expression pattern, originally showing a cytoplasmatic along with a membrane staining by IHC. While GD2 has been reported to be predominately exposed on the cell membrane, GD2 pattern in BC is coherent with results reported in other neuroectodermal and mesenchymal neoplasms [12, 20, 27] and it can be due to the biosynthetic pathway of the ganglioside.

Our cohort of BC was heterogeneous by age at diagnosis, histotype, grading, Ki-67, tumor size and receptor phenotype. Student's *t-test*, Fisher's exact test and multivariate logistic regression analysis did not demonstrate any statistically significant correlation between GD2 expression and any of these variables, including Ki-67. The age appeared as a discriminant factor with older patients having a greater GD2 level, however this finding may be biased by the fact that most of OUR metasplastic BC samples falls into an advanced age. In this sense a larger patient cohort shall be requested to further validate these findings. Interestingly, the univariate logistic regression analysis showed a statistically significant correlation of ganglioside GD2 with triple-negative BC in comparison to ER/PR +/HER2 –, thus confirming the preliminary data in a single case of TNBC reported [14]. A similar trend was found with metaplastic carcinomas compared to IDC, although not statistically significant.

Some considerations have to be shared about GD2 expression pattern according to the histotype. Despite the limited number of patients included in the study, the majority of metaplastic breast carcinoma samples were GD2 positive, showing the strongest staining in their spindle-like elements and not in their epithelial-like component. The expression of BCSC-related antigens and EMT markers (Zeb-1, E-cadherin) has already been demonstrated in metaplastic BC histological specimens, where the positivity was mostly related to the non-epithelial/mesenchymal part of these tumors [33]. BCSC biomarkers, including GD2 in association with ALDH, were also described in a cohort of phyllodes tumors, a rare subtype of breast neoplasm that shows some affinities with metaplastic tumors [34]. We can speculate that GD2 expression in the BC specimens analyzed in this study might be related to BCSC and to the activation of the process of EMT. We also suggest that GD2-positive specimens contain cells that are undergoing EMT and the association of GD2 with the triple-receptor negative phenotype along with the constant expression of this antigen in the metaplastic BC specimens seems to corroborate this. These neoplasms are in fact characterized by a structural mesenchymal component [33, 35] and might exhibit a more constitutive and regular expression of BCSC and EMT biomarkers, including GD2.

This investigation retains limitations mostly due to the small sample size and to the sampling method. However, the study had an explorative intent and was performed since no consistent data on GD2 and its correlation with clinical-pathological parameters in BC have been previously reported. Another limitation of the study is the lack of an external validation of GD2 IHC staining procedures and scoring system. In this case a relevant effort was dedicated to identify an anti-GD2 antibody capable to generate consistent results on paraffin embedded BC specimens. The proposed scoring system has now to be challenged in a larger cohort of patients in the effort to generate a standardized method for GD2 immunohistochemical evaluation in BC and, possibly, in other cancer types.

To conclude, GD2 staining was for the first time described in a heterogeneous cohort of BC specimens, showing a possible correlation with histotypes strongly associated with EMT. Although studies are now needed to consolidate these results, GD2 appears as a new biomarker in BC, in particular for the TNBC variants, which are still orphans of specific markers for diagnostics and possible targeted therapies.

## MATERIALS AND METHODS

### Patients and specimens

The archive of the Pathology Division of Modena University Hospital and Padua University Hospital was searched to identify sixty-three female patients diagnosed with infiltrating BC from 2001 to 2014, with available samples from primary tumors (mastectomy or quadrantectomy specimens). Clinical-pathological data included age at diagnosis, tumor size (T), histological type, histological grade (according to the Nottingham Grading System [36]), Ki-67, Estrogen receptor, Progesterone receptor and HER2 status as described in the pathological report and confirmed by the pathologist's review. As stated in the latest ASCO/CAP guidelines [37], hormone receptors were considered positive when ≥ 1% of cancer cells stained by immunohistochemistry (IHC). HER2 was assessed as over-expressed in those cases showing ISH positive, or IHC score 3+, or IHC score 2+ and ISH positive (ASCO/CAP guidelines 2013 [38]). Prior treatment with chemotherapy or radiotherapy was an exclusion criterion. Written informed consent was obtained from alive patients. According to these inclusion and exclusion criteria, we selected a cohort of 51 BC specimens for a preliminary evaluation of GD2 staining. After observing a relevant expression of the antigen in three samples of metaplastic BC included in this initial dataset, we enriched our population with 12 more cases of metaplastic BC, so that a final cohort of 63 patients was considered. The study protocol was approved by the Ethical Committees of Modena and Padua.

### Immunohistochemistry

Preliminary IHC tests with different anti-GD2 antibodies (Table 4) were performed on neuroblastoma and melanoma histological specimens and cell lines (data not shown), in order to set up the procedure. After extensive testing, the selected antibody was an anti-GD2 rabbit polyclonal antibody (1:200 dilution; Matreya LLC, Pleasant Gap, PA). Formalin-fixed, paraffin-embedded 4-μm-thick tissue sections from BC specimens were transferred to microscope slides. Sections were dried overnight at 40°C; after heat-induced antigen retrieval, slides were incubated with the anti-GD2 antibody for 12 minutes at 37°C, using an automatic immunohistochemical staining device (Benchmark XT; Ventana Medical Systems, Tucson, AZ). The reaction was subsequently detected with the appropriate reagent from Kit Ventana DAB (Ventana Medical Systems, Tucson, AZ). Lastly, sections were counterstained with Harris hematoxylin and analyzed by standard light microscope (Axio Imager M2 - Zeiss) with Plan-Apochromat 20X/0.8, EC Plan-Neofluar 40X/0.75 and EC Plan-Neofluar 63X/1.25-Oil objectives. Slides stained with no primary antibody were used as negative control. Photomicrographs were acquired with Axiocam 506 color camera (software ZEN pro - Zeiss).

**Table 4:** Anti-GD2 antibodies tested for immunohistochemistry

Name	Type	Source	Dilution	Company
Anti-ganglioside GD2	Polyclonal, isotype IgG/IgM	Rabbit	1:200	*Matreya LLC*
Anti-ganglioside GD2 antibody (2Q549)	Monoclonal, IgG_2a_	Mouse	1:10; 1:40	*Abcam*
Ganglioside GD2 (14G2a)	Monoclonal, IgG_2a_	Mouse	1:25; 1:50	*Santa Cruz Biotechnology*

### Scoring

Any distinct positive IHC staining of BC cells within the samples was regarded as positive GD2 expression, with a modified scoring system from Ziebarth et al. there applied in uterine leiomyosarcoma [27]. With the aim to properly quantify GD2 expression in the considered specimens, we established a semi-quantitative scoring system, based on three different levels of staining intensity: 0 (negative), 1+ (low positivity), 2+ (high positivity). In those samples having either 1+ or 2+ score, the portion of GD2 positive tumor cells was expressed as percentage (%).

### Statistical analysis

The primary study endpoint was the evaluation of GD2 expression and its correlation with clinical-pathological features in a cohort of BC patients. Given the explorative aim of this investigation, no sample size calculation was performed. Relationships between the BC clinical-pathological parameters and GD2 expression were analyzed using χ^2^ test and Fisher's exact test for categorical variables; Student's *t-test* was used for continuous variables. Furthermore, univariate and multivariate logistic regression analyses were performed. The significance level was set at *p <* 0.05. Data were analyzed using STATA13 (StataCorp. 2011. Stata: Release 12. Statistical Software. College Station, TX: StataCorp LP).

## Abbreviations

BC: breast cancer
TNBC: triple-negative breast cancer
MSC: mesenchymal stromal cells
BCSC: breast cancer stem cells
EMT: epithelial-mesenchymal transition
IHC: immunohistochemistry
IDC: invasive ductal carcinoma
NOS: not otherwise specified
ILC: invasive lobular carcinoma

## References

[1] Gradishar WJ, Anderson BO, Balassanian R, Blair SL, Burstein HJ, Cyr A, Elias AD, Farrar WB, Forero A, Giordano SH, Goetz M, Goldstein LJ, Hudis CA (2015). Breast Cancer, Version 1.2016. J Natl Compr Canc Netw.

[2] Prat A, Karginova O, Parker JS, Fan C, He X, Bixby L, Harrell JC, Roman E, Adamo B, Troester M, Perou CM (2013). Characterization of cell lines derived from breast cancers and normal mammary tissues for the study of the intrinsic molecular subtypes. Breast Cancer Res Treat.

[3] Perou CM, Sorlie T, Eisen MB, van de Rijn M, Jeffrey SS, Rees CA, Pollack JR, Ross DT, Johnsen H, Akslen LA, Fluge O, Pergamenschikov A, Williams C (2000). Molecular portraits of human breast tumours. Nature.

[4] Prat A, Perou CM (2011). Deconstructing the molecular portraits of breast cancer. Mol Oncol.

[5] Prat A, Parker JS, Karginova O, Fan C, Livasy C, Herschkowitz JI, He X, Perou CM (2010). Phenotypic and molecular characterization of the claudin-low intrinsic subtype of breast cancer. Breast Cancer Res.

[6] Lehmann BD, Bauer JA, Chen X, Sanders ME, Chakravarthy AB, Shyr Y, Pietenpol JA (2011). Identification of human triple-negative breast cancer subtypes and preclinical models for selection of targeted therapies. J Clin Invest.

[7] Navid F, Santana VM, Barfield RC (2010). Anti-GD2 antibody therapy for GD2-expressing tumors. Curr Cancer Drug Targets.

[8] Martinez C, Hofmann TJ, Marino R, Dominici M, Horwitz EM (2007). Human bone marrow mesenchymal stromal cells express the neural ganglioside GD2: a novel surface marker for the identification of MSCs. Blood.

[9] Kohla G, Stockfleth E, Schauer R (2002). Gangliosides with O-acetylated sialic acids in tumors of neuroectodermal origin. Neurochem Res.

[10] Thurin J, Thurin M, Herlyn M, Elder DE, Steplewski Z, Clark WH, Koprowski H (1986). GD2 ganglioside biosynthesis is a distinct biochemical event in human melanoma tumor progression. FEBS Lett.

[11] Chang HR, Cordon-Cardo C, Houghton AN, Cheung NK, Brennan MF (1992). Expression of disialogangliosides GD2 and GD3 on human soft tissue sarcomas. Cancer.

[12] Kailayangiri S, Altvater B, Meltzer J, Pscherer S, Luecke A, Dierkes C, Titze U, Leuchte K, Landmeier S, Hotfilder M, Dirksen U, Hardes J, Gosheger G (2012). The ganglioside antigen G(D2) is surface-expressed in Ewing sarcoma and allows for MHC-independent immune targeting. Br J Cancer.

[13] Long AH, Highfill SL, Cui Y, Smith JP, Walker AJ, Ramakrishna S, El-Etriby R, Galli S, Tsokos M, Orentas RJ, Mackall CL (2016). Reduction of MDSCs with all-trans retinoic acid improves CAR therapy efficacy for sarcomas. Cancer Immunol Res.

[14] Battula VL, Shi Y, Evans KW, Wang RY, Spaeth EL, Jacamo RO, Guerra R, Sahin AA, Marini FC, Hortobagyi G, Mani SA, Andreeff M (2012). Ganglioside GD2 identifies breast cancer stem cells and promotes tumorigenesis. J Clin Invest.

[15] Liang YJ, Ding Y, Levery SB, Lobaton M, Handa K, Hakomori SI (2013). Differential expression profiles of glycosphingolipids in human breast cancer stem cells vs. cancer non-stem cells. Proc Natl Acad Sci USA.

[16] Sarkar TR, Battula VL, Werden SJ, Vijay GV, Ramirez-Pena EQ, Taube JH, Chang JT, Miura N, Porter W, Sphyris N, Andreeff M, Mani SA (2015). GD3 synthase regulates epithelial-mesenchymal transition and metastasis in breast cancer. Oncogene.

[17] Yu RK, Tsai YT, Ariga T, Yanagisawa M (2011). Structures, biosynthesis, and functions of gangliosides—an overview. J Oleo Sci.

[18] Birkle S, Zeng G, Gao L, Yu RK, Aubry J (2003). Role of tumor-associated gangliosides in cancer progression. Biochimie.

[19] Ledeen R, Wu G (2011). New findings on nuclear gangliosides: overview on metabolism and function. J Neurochem.

[20] Schulz G, Cheresh DA, Varki NM, Yu A, Staffileno LK, Reisfeld RA (1984). Detection of ganglioside GD2 in tumor tissues and sera of neuroblastoma patients. Cancer Res.

[21] Sariola H, Terava H, Rapola J, Saarinen UM (1991). Cell-surface ganglioside GD2 in the immunohistochemical detection and differential diagnosis of neuroblastoma. Am J Clin Pathol.

[22] Prapa M, Caldrer S, Spano C, Bestagno M, Golinelli G, Grisendi G, Petrachi T, Conte P, Horwitz EM, Campana D, Paolucci P, Dominici M (2015). A novel anti-GD2/4–1BB chimeric antigen receptor triggers neuroblastoma cell killing. Oncotarget.

[23] Tsuchida T, Saxton RE, Morton DL, Irie RF (1987). Gangliosides of human melanoma. J Natl Cancer Inst.

[24] Cheresh DA, Rosenberg J, Mujoo K, Hirschowitz L, Reisfeld RA (1986). Biosynthesis and expression of the disialoganglioside GD2, a relevant target antigen on small cell lung carcinoma for monoclonal antibody-mediated cytolysis. Cancer Res.

[25] Mennel HD, Bosslet K, Geissel H, Bauer BL (2000). Immunohistochemically visualized localisation of gangliosides Glac2 (GD3) and Gtri2 (GD2) in cells of human intracranial tumors. Exp Toxicol Pathol.

[26] Heiner JP, Miraldi F, Kallick S, Makley J, Neely J, Smith-Mensah WH, Cheung NK (1987). Localization of GD2-specific monoclonal antibody 3F8 in human osteosarcoma. Cancer Res.

[27] Ziebarth AJ, Felder MA, Harter J, Connor JP (2012). Uterine leiomyosarcoma diffusely express disialoganglioside GD2 and bind the therapeutic immunocytokine 14.18-IL2: implications for immunotherapy. Cancer Immunol Immunother.

[28] Creighton CJ, Chang JC, Rosen JM (2010). Epithelial-mesenchymal transition (EMT) in tumor-initiating cells and its clinical implications in breast cancer. J Mammary Gland Biol Neoplasia.

[29] May CD, Sphyris N, Evans KW, Werden SJ, Guo W, Mani SA (2011). Epithelial-mesenchymal transition and cancer stem cells: a dangerously dynamic duo in breast cancer progression. Breast Cancer Res.

[30] Creighton CJ, Li X, Landis M, Dixon JM, Neumeister VM, Sjolund A, Rimm DL, Wong H, Rodriguez A, Herschkowitz JI, Fan C, Zhang X, He X (2009). Residual breast cancers after conventional therapy display mesenchymal as well as tumor-initiating features. Proc Natl Acad Sci USA.

[31] Battula VL, Evans KW, Hollier BG, Shi Y, Marini FC, Ayyanan A, Wang RY, Brisken C, Guerra R, Andreeff M, Mani SA (2010). Epithelial-mesenchymal transition-derived cells exhibit multilineage differentiation potential similar to mesenchymal stem cells. Stem cells.

[32] De Giorgi U, Cohen EN, Gao H, Mego M, Lee BN, Lodhi A, Cristofanilli M, Lucci A, Reuben JM (2011). Mesenchymal stem cells expressing GD2 and CD271 correlate with breast cancer-initiating cells in bone marrow. Cancer Biol Ther.

[33] Zhang Y, Toy KA, Kleer CG (2012). Metaplastic breast carcinomas are enriched in markers of tumor-initiating cells and epithelial to mesenchymal transition. Mod Pathol.

[34] Lin JJ, Huang CS, Yu J, Liao GS, Lien HC, Hung JT, Lin RJ, Chou FP, Yeh KT, Yu AL (2014). Malignant phyllodes tumors display mesenchymal stem cell features and aldehyde dehydrogenase/disialoganglioside identify their tumor stem cells. Breast Cancer Res.

[35] Jeong H, Ryu YJ, An J, Lee Y, Kim A (2012). Epithelial-mesenchymal transition in breast cancer correlates with high histological grade and triple-negative phenotype. Histopathology.

[36] Rakha EA, Reis-Filho JS, Baehner F, Dabbs DJ, Decker T, Eusebi V, Fox SB, Ichihara S, Jacquemier J, Lakhani SR, Palacios J, Richardson AL, Schnitt SJ (2010). Breast cancer prognostic classification in the molecular era: the role of histological grade. Breast Cancer Res.

[37] Hammond ME, Hayes DF, Dowsett M, Allred DC, Hagerty KL, Badve S, Fitzgibbons PL, Francis G, Goldstein NS, Hayes M, Hicks DG, Lester S, Love R (2010). American Society of Clinical Oncology/College Of American Pathologists guideline recommendations for immunohistochemical testing of estrogen and progesterone receptors in breast cancer. J Clin Oncol.

[38] Wolff AC, Hammond ME, Hicks DG, Dowsett M, McShane LM, Allison KH, Allred DC, Bartlett JM, Bilous M, Fitzgibbons P, Hanna W, Jenkins RB, Mangu PB (2013). Recommendations for human epidermal growth factor receptor 2 testing in breast cancer: American Society of Clinical Oncology/College of American Pathologists clinical practice guideline update. J Clin Oncol.

